# Potential Roles of Flavonoids from Chrysanthemi Flos and Chrysanthemi Indici Flos in Modulating Bile Acid Metabolism: Mechanistic Insights and Future Perspectives

**DOI:** 10.3390/ijms27209164

**Published:** 2026-10-16

**Authors:** Shuhui Zhao, Ling Zeng, Heng Tang, Yuxuan Cui, Runye Zeng, Jingchen Xie

**Affiliations:** 1 School of Pharmacy, Hunan University of Chinese Medicine, Changsha 410208, China; zhaoshuhui0523@163.com (S.Z.); 19313189791@163.com (L.Z.); tanghengyy@outlook.com (H.T.); 19313189039@163.com (Y.C.); 17573888760@163.com (R.Z.); 2 Key Laboratory for Quality Evaluation of Bulk Herbs of Hunan Province, Hunan University of Chinese Medicine, Changsha 410208, China

**Keywords:** Chrysanthemi Flos, Chrysanthemi Indici Flos, flavonoids, bile acid metabolism, liver injury, choleretic, hepatoprotective effects

## Abstract

Changes in bile acid metabolism are associated with metabolic liver disease, inflammation, and immune dysfunction. Therefore, regulating bile acid homeostasis may help in preventing and treating liver injury and metabolic disorders. Chrysanthemi Flos and Chrysanthemi Indici Flos contain many bioactive flavonoids and have several reported pharmacological effects. However, how their flavonoids regulate bile acid metabolism has not been reviewed in detail. This review summarizes 52 flavonoids identified in Chrysanthemi Flos and Chrysanthemi Indici Flos. We review their reported effects on bile acid conjugation and synthesis, secretion and excretion, enterohepatic circulation, and main receptors, including FXR (farnesoid X receptor, nuclear bile acid receptor; regulates synthesis/transport), TGR5 (Takeda G protein-coupled receptor 5, GPCR (G protein-coupled receptor) bile acid receptor; GLP-1 (glucagon-like peptide-1) secretion, energy/anti-inflammation, incretin; glucose/lipid homeostasis), and LXRα (liver X receptor α, nuclear receptor; cholesterol efflux and CYP7A1 (cholesterol 7α-hydroxylase, rate-limiting enzyme of classical bile acid synthesis; cholesterol → 7α-hydroxycholesterol) regulation). We also consider how these pathways may affect inflammation and metabolic disorders. This review relates the reported mechanisms to the traditional use of these medicinal plants for “clearing the liver and promoting bile flow.” It also outlines research needs for assessing natural hepatoprotective and metabolic disease interventions that target bile acid metabolism.

## 1. Introduction

Bile acids are the primary products of cholesterol metabolism in the liver and play key roles in fat absorption and the maintenance of cholesterol homeostasis. In recent years, as research has progressed, accumulating evidence has indicated that bile acid metabolism contributes to digestion and regulates systemic energy metabolism, immune responses, and inflammatory reactions. As a result, it influences the onset and progression of various diseases, such as metabolic disorders including obesity, type 2 diabetes, and nonalcoholic fatty liver disease [1], as well as cancer [2] and atherosclerosis [3].

Evidence increasingly links flavonoids and the regulation of bile acid metabolism. Studies indicate that Hyperoside [4], Luteolin [5], and whole-grain oat flavonoids [6], among others, may ameliorate conditions such as cholestatic liver injury, nonalcoholic fatty liver disease (NAFLD), and hyperlipidemia by changing bile acid metabolism. More importantly, flavonoids are the representative bioactive compounds in Chrysanthemi Flos (the dried capitulum of *Chrysanthemum morifolium *Ramat.) and Chrysanthemi Indici Flos (the dried capitulum of *Chrysanthemum indicum* L.), both belonging to the Asteraceae family. As traditional Chinese medicinal materials used both medicinally and as herbal teas, they possess many pharmacological effects, including antibacterial, anti-inflammatory, antioxidant, vasodilatory, hypoglycemic, lipid-lowering, antihypertensive, and anti-tumor effects [7,8,9]. Modern pharmacological studies indicate that their medicinal value stems from various bioactive components, including essential oils, flavonoids, triterpenes, chlorogenic acid, and amino acids [1,10]. Among these, flavonoids are considered one of the most important active components responsible for their pharmacological effects. Recent studies have demonstrated that specific flavonoids from these plants directly regulate bile acid metabolism. Total flavonoids from Chrysanthemi Flos upregulate CYP7A activity to promote cholesterol-to-bile acid conversion in hyperlipidemic rats [11]. In addition, the major flavonoids in Chrysanthemi Flos and Chrysanthemi Indici Flos have been reported to regulate bile acid metabolism. Luteolin and Luteoloside significantly upregulate CYP7A1 in hyperlipidemic models [11,12]. Apigenin restores bile acid secretion/excretion balance in DDC (3,5-diethoxycarbonyl-1,4-dihydrocollidine)-induced cholestatic mice [13]. Linarin significantly alters hepatic bile acid metabolic genes and metabolites via FXR/TGR5 pathways [14]. Rutin alleviates BDL-induced cholestatic liver injury [15].

However, there is currently a lack of a systematic summary of the mechanisms by which flavonoids in Chrysanthemi Flos and Chrysanthemi Indici Flos influence bile acid metabolism, which hinders assessment of their potential for clinical use. Therefore, to assess this evidence, the authors followed the PRISMA (Preferred Reporting Items for Systematic Reviews and Meta-Analyses) guidelines and conducted a search using keywords such as “Chrysanthemi Flos,” “Chrysanthemi Indici Flos,” “flavone,” and “bile acid metabolism” in multiple literature databases, including China National Knowledge Infrastructure (CNKI), PubMed, and Web of Science. Based on the search results, we organized and reviewed the molecular mechanisms by which the flavonoid components of Chrysanthemi Flos and Chrysanthemi Indici Flos produce their reported effects by regulating bile acid metabolism. This review aims to provide new insights and strategies for the prevention and treatment of related diseases, thus promoting the further investigation and possible clinical use of the medicinal value of Chrysanthemi Flos and Chrysanthemi Indici Flos.

## 2. Flavonoids in Chrysanthemi Flos and Chrysanthemi Indici Flos

**Figure 1:**
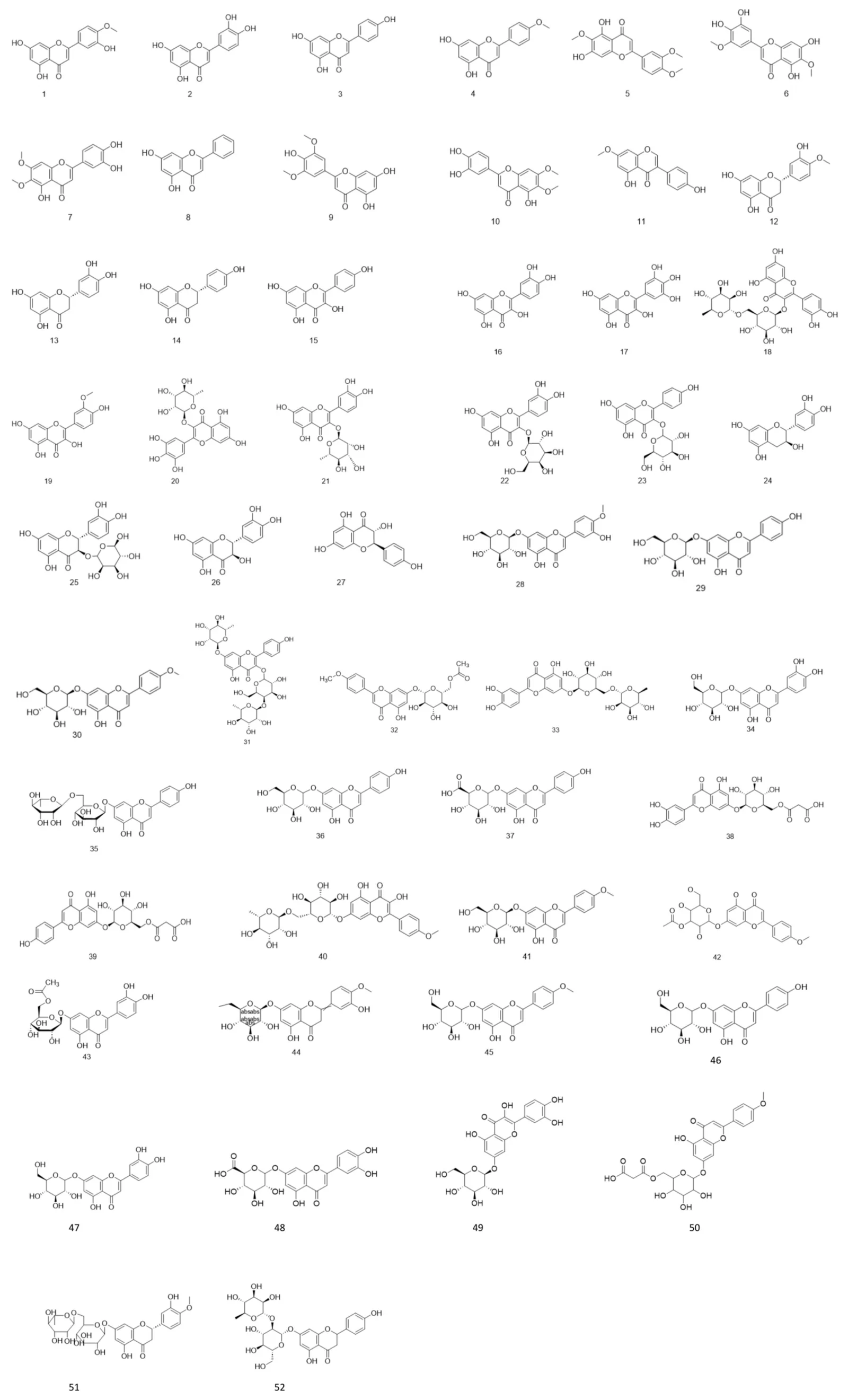
Structures of flavonoids in Chrysanthemi Flos and Chrysanthemi Indici Flos. This figure displays the chemical structures of 52 flavonoids identified in these two plants, categorized into flavones, flavonols, flavanones, isoflavones, catechins, and their glycosides, providing a comprehensive overview of their structural diversity.

Flavonoids are associated with the pharmacological effects of Chrysanthemi Flos and Chrysanthemi Indici Flos and are the primary active components in these plants. Current research has identified a total of 52 flavonoids in Chrysanthemi Indici Flos and Chrysanthemi Flos, of which 16 are found in Chrysanthemi Indici Flos, while 38 are found in Chrysanthemi Flos. These are mainly classified as flavonoids, isoflavonoids, dihydroflavonoids, flavonols, catechins, dihydroflavonols, flavonoid glycosides, isoflavonoid glycosides, and dihydroflavonoid glycosides. For specific compounds, structures, and distribution details, see Table 1 and Figure 1.

## 3. Bile Acid Metabolism and Enterohepatic Circulation

### 3.1. Bile Acid Synthesis: Classical and Alternative Pathways

**Figure 2:**
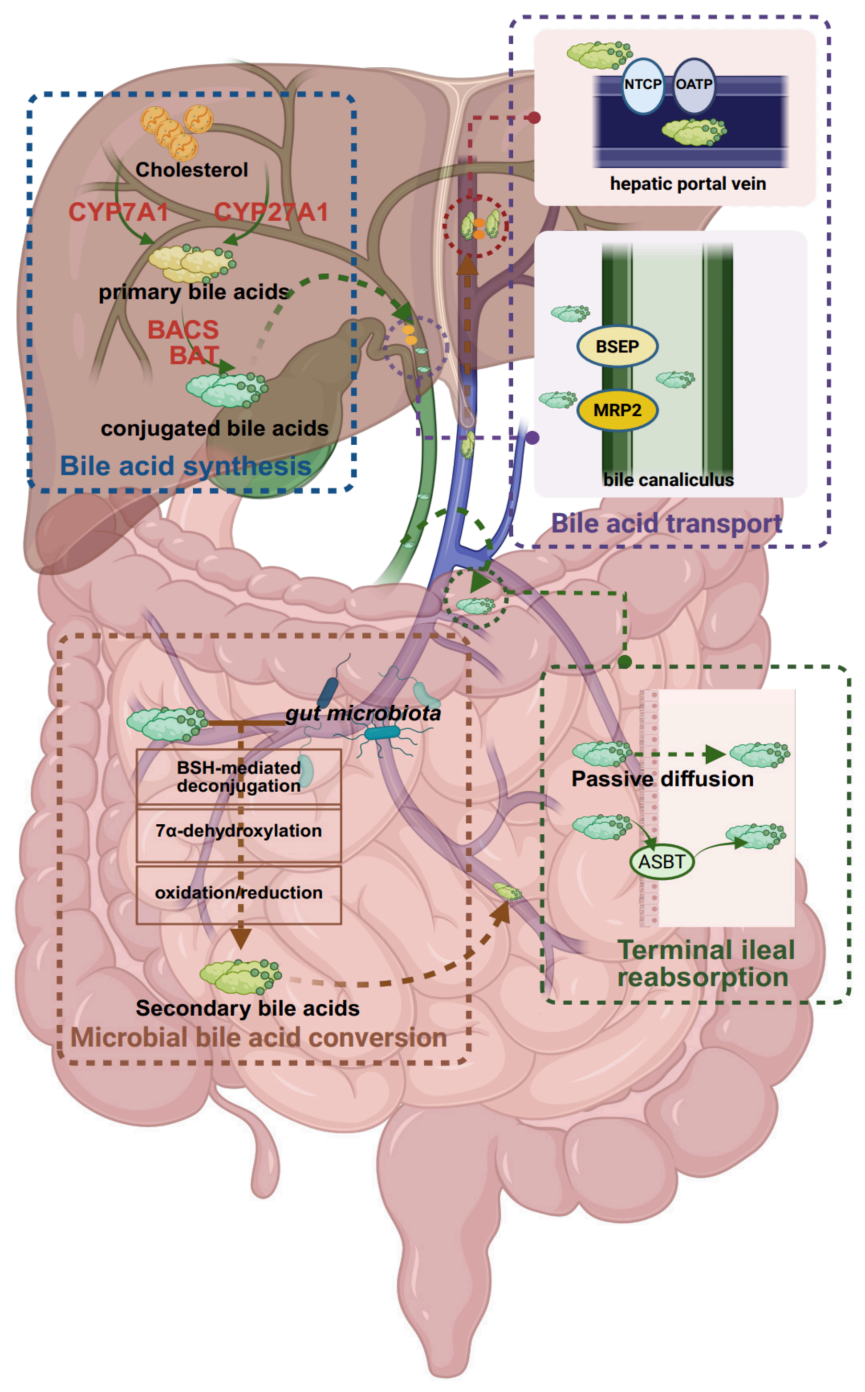
Bile acid metabolism and enterohepatic circulation. Created in BioRender. Jingchen, X. (2026) https://BioRender.com/fh42hu5. This figure summarizes the classical and alternative pathways of bile acid synthesis, conjugation, transport, and enterohepatic circulation, highlighting key enzymes (e.g., CYP7A1, CYP27A1) and transporters (e.g., BSEP, NTCP, ASBT, MRP2) involved in maintaining bile acid homeostasis.

The synthesis of bile acids begins in hepatocytes, using cholesterol as a precursor, and proceeds mainly through two pathways (Figure 2): (1) the classical pathway (neutral pathway). CYP7A1 is the main rate-limiting enzyme in the classical pathway of cholesterol conversion to bile acids. This enzyme is tissue-specific, expressed and exerting its catalytic function exclusively in liver microsomes [28]. CYP7A1 first specifically catalyzes the hydroxylation of cholesterol at the 7α-position to produce 7α-hydroxycholesterol.

This is followed by a series of enzymatic reactions, including sterol nucleus reduction, additional hydroxylation, and side-chain oxidative cleavage, finally yielding two primary bile acids, CA (cholic acid) and CDCA (chenodeoxycholic acid) [29]. This pathway is the primary metabolic route for bile acid synthesis in humans, accounting for about 75% of total bile acid synthesis [30]. (2) Alternative pathway (acidic pathway). CYP27A1 (Cholesterol 27α-hydroxylase, Initiates alternative bile acid synthesis; cholesterol → 27-hydroxycholesterol) is the main enzyme that initiates the alternative pathway (also known as the acidic pathway) for bile acid synthesis. This enzyme is widely distributed in the mitochondria of various tissue cells, including the liver and kidneys [28]. In this pathway, CYP27A1 first catalyzes the hydroxylation of cholesterol at the C27 position to form 27-hydroxycholesterol. This intermediate then undergoes a series of subsequent modification reactions, finally leading mainly to the formation of CDCA [29]. When the classical pathway is blocked, this alternative pathway can be compensatorily enhanced, thus maintaining the overall stability of bile acid synthesis and metabolism in vivo [30].

### 3.2. Bile Acid Conjugation and Transport

The synthesized unconjugated bile acids undergo a conjugation reaction to enhance their water solubility and are then secreted into the bile through transport proteins (Figure 2).

#### 3.2.1. Conjugation Reactions

In the formation of conjugated bile acids, there are two main enzymes that act sequentially: BACS (bile acid-CoA synthase, activates bile acids to bile acid-CoA) and BAT (bile acid-CoA amino acid N-acyltransferase, conjugates bile acid-CoA with glycine/taurine). BACS first catalyzes the activation of unconjugated bile acids with coenzyme A (CoA) to form bile acid-CoA. Then, BAT transfers the bile acid moiety from the acyl-CoA to the amino group of glycine or taurine, thus completing the covalent binding. The resulting products, such as glycocholate and taurocholate, are common conjugated bile acids [29]. This conjugation process increases the polarity of bile acids, which reduces their cytotoxicity and facilitates their excretion via the biliary tract, thus completing a key step in the enterohepatic circulation of bile acids.

#### 3.2.2. Intrahepatic Transport

In the hepatic excretion phase of the enterohepatic circulation of bile acids, conjugated bile acids must be actively secreted into the bile via specific transporters located on the canalicular membranes of hepatocytes. This process is mainly mediated by the BSEP(ATP-dependent canalicular bile acid export), which is responsible for transporting bile acids against a concentration gradient. Also, MRP2 (multidrug resistance-associated protein 2, canalicular efflux of conjugated bile acids/organic anions) also participates in the excretion of some bile acids and their conjugates [28].

### 3.3. Enterohepatic Circulation of Bile Acids

The enterohepatic circulation is a central process for the efficient reutilization of bile acids, involving reabsorption in the intestine and reuptake in the liver (Figure 2).

#### 3.3.1. Intestinal Reabsorption

The reabsorption of bile acids in the intestine occurs mainly in the terminal ileum. This process involves two distinct absorption mechanisms: conjugated bile acids are efficiently reabsorbed mainly through active transport mediated by the ASBT (apical sodium-dependent bile acid transporter, Ileal apical Na^+^-dependent bile acid reabsorption) on the apical membrane of enterocytes, whereas unconjugated bile acids may enter enterocytes by passive diffusion due to their high lipid solubility. Through these pathways, about 95% of intestinal bile acids are reabsorbed and then returned to the liver via the portal venous system to be taken up again and utilized, thus constituting the core component of the enterohepatic circulation of bile acids [31].

#### 3.3.2. Hepatic Reuptake and Resecretion

In the intrahepatic phase of the enterohepatic circulation, bile acids reaching the liver via the portal venous system must first be efficiently taken up by hepatocytes. This process is mainly mediated by two specific transporters located on the basolateral membrane of hepatocytes: The NTCP (Na^+^-taurocholate cotransporting polypeptide, hepatic basolateral Na^+^-dependent conjugated bile acid uptake) is mainly responsible for the sodium-dependent uptake of conjugated bile acids, while the members of the OATP (organic anion transporting polypeptide, hepatic basolateral Na^+^-independent bile acid uptake) family, which mainly mediate the sodium-independent uptake of free and some conjugated bile acids [29].

The reabsorbed bile acids then mix with newly synthesized conjugated bile acids within the hepatocytes. Then, these endogenous and recycled bile acids are actively secreted into the bile canaliculi via transport systems such as the BSEP located on the canalicular membrane, re-entering the bile. This “uptake-resecretion” process constitutes a main stage of the enterohepatic circulation within the liver, ensuring the efficient recycling of bile acids [31].

### 3.4. Intestinal Biotransformation and Effects of the Gut Microbiota

Bile acids undergo microbially mediated structural modifications in the intestine, resulting in the formation of secondary bile acids (Figure 2).

#### 3.4.1. Bacterial Transformation

The metabolism of bile acids by gut microbiota is an important step in the enterohepatic circulation, mainly occurring through three principal routes. First, in the hydrolysis and deconjugation step, intestinal bacteria (such as *Lactobacillus* and *Bifidobacterium*) secrete bile salt hydrolases. These enzymes specifically hydrolyze the amide bonds of conjugated bile acids (such as glycocholic acid and taurocholic acid), converting them into unconjugated bile acids. This is a prerequisite for subsequent microbial modifications [32]. Second, 7α-dehydroxylation is mainly catalyzed by specific anaerobic bacteria in the intestine (such as *Clostridium* spp.); this reaction removes the 7α-hydroxyl group from primary bile acids. For example, this process converts cholic acid to DCA (deoxycholic acid) and chenodeoxycholic acid to LCA (lithocholic acid), yielding secondary bile acids with different physiological and pathological significance [30]. Also, microorganisms may further alter the structure of bile acids through other enzymatic modifications. For example, hydroxysteroid dehydrogenase can catalyze the oxidation, reduction, or epimerization of bile acid hydroxyl groups, resulting in several structurally distinct bile acid derivative metabolites [29].

#### 3.4.2. Interactions Between the Gut Microbiota and Bile Acids

Bile acids and the gut microbiota form a complex bidirectional regulatory system, in which they influence each other and maintain a balance. First, bile acids directly affect the gut microbiota. For example, secondary bile acids such as DCA possess surfactant properties that may disrupt the cell membranes of specific bacteria (such as certain Gram-positive bacteria), thus inhibiting their growth and shaping the composition and structure of the gut microbiota [33]. Conversely, the gut microbiota influences the composition and properties of the bile acid pool through extensive biotransformation. As mentioned earlier, microbiota converts primary bile acids into structurally diverse secondary bile acids and their derivatives through various enzymatic reactions, such as hydrolysis, dehydroxylation, and oxidation, which determine the metabolic profile of bile acids in the enterohepatic circulation [29]. These secondary bile acids derived from the microbiota (such as DCA and LCA) serve as important signaling molecules. They act as endogenous ligands for the FXR and the G protein-coupled bile acid receptor TGR5 in host cells. Through these receptors, secondary bile acids regulate several physiological processes systemically, including glucose and lipid metabolism, energy expenditure, and immune and inflammatory responses in the gut and liver, thus influencing the host’s metabolic health and immune balance [6].

## 4. The Mechanism of Flavonoids from Chrysanthemi Flos and Chrysanthemi Indici Flos Regulating Bile Acids

### 4.1. Bile Acid Conjugation and Synthesis

Flavonoids act on main proteins involved in bile acid synthesis and conjugation, thus regulating the homeostasis of bile acid metabolism. Experimental studies have shown that linarin (the main component of Chrysanthemi Indici Flos) promotes cholesterol excretion by upregulating ABCG5/ABCG8 (ATP-binding cassette sub-family G member5/ATP-binding cassette sub-family G member8, Exports cholesterol/plant sterols into bile; limits absorption) and inhibits CYP7A1, and may improve bile acid metabolism [34]. Quercitrin may promote the conversion of cholesterol to bile acids by targeting the mTOR (mechanistic target of rapamycin, kinase; upstream of YY1/CYP7A1)-mediated YY1 (Yin Yang 1, a multifunctional transcription factor that regulates CYP7A1 expression)/CYP7A1 signaling pathway, thus reducing hepatic lipid accumulation [35]. It may also enhance the expression of FXR1 and CYP7A1 in liver tissue, promoting the metabolism of cholesterol into bile acids and alleviating hyperlipidemia in mice [36]. When combined with capsaicin, it may upregulate the expression levels of FXR1 and FGF19 (fibroblast growth factor 19), accelerate cholesterol breakdown, promote the conversion of cholesterol to bile acids, and alleviate lipid accumulation in the HepG2 high-fat cell model [37]. Also, FGFit has been studied as a treatment for hyperlipidemia (HLP) by upregulating CYP7A1 mRNA to promote cholesterol transport [38]. Quercetin supplementation may upregulate hepatic CYP7A1 expression and activity, thereby promoting cholesterol-to-bile acid conversion in the liver and contributing to cholesterol homeostasis [39]. Also, in a diet-induced obesity mouse model, it reshapes the overall structure of the microbiome and improve obesity by synergistically regulating host bile acid metabolism through the ILA/FTO (Fat mass and obesity-associated)/m6A (N6-methyladenosine)/CYP8B1 (sterol 12α-hydroxylase, 12α-Hydroxylase; promotes CA synthesis)/CA axis [40]. In terms of protection against atherosclerosis, it may upregulate CYP27A1 protein expression, inhibit CD36-mediated cholesterol uptake, and increase cholesterol efflux mediated by the LXRα-ABCA1 (ATP-binding cassette sub-family A member 1, Cholesterol efflux to apoA-I; HDL formation)/G1 (ATP-binding cassette sub-family G member 1) signaling pathway, reducing the level of macrophage foam cells formation in vitro [41]. In emodin-injured L02 cells, hyperoside reduced CYP7A1 mRNA and protein expression while improving cell viability [42]. In ApcMin/+ mice, kaempferol increased CYP27A1 and CYP8B1 expression and restored reduced CDCA and 12α-hydroxylated bile acid levels, alongside lower tumor burden [43]. Also in high-fat-diet-induced nonalcoholic steatohepatitis mice, it increased hepatic CYP27A1 and NTCP mRNA and altered bile acid composition [44]. Studies have shown that kaempferol and isorhamnetin increase CYP7A1 mRNA expression and reduce lipid deposition in zebrafish and HepaRG cells [45]. Hesperidin reduces bile acid synthesis by activating FXR and inhibiting CYP7A1 transcription, thus alleviating constipation in a mouse model [46]. By activating FXR, it promotes bile acid secretion from the liver and inhibits its uptake and synthesis in the liver, thus exerting a protective effect against ANIT-induced cholestatic liver injury in mice [47]. Naringin promotes bile acid synthesis by upregulating CYP7A1 via suppression of the FXR/FGF15 pathway and facilitates the accumulation of conjugated bile acids, including tauro-conjugated bile acids. In atherosclerosis models, naringin significantly enhances cholesterol-to-bile acid conversion and promotes the generation and excretion of conjugated bile acids [48]. Myricetin and myricitrin exhibit good lipid-lowering activity in C57BL/6j mice with hyperlipidemia by upregulating CYP7A1 expression and increasing bile acid concentrations [49]. Catechins promote the conversion of cholesterol to bile acids by inhibiting the feedback regulation of CYP7A1 mediated by the intestinal FXR/FGF15 signaling pathway [50]. In the hyperlipidemic rodent model, luteolin increased hepatic Cyp7a1 mRNA relative to the model control, supporting enhanced cholesterol-to-bile acid conversion as a proposed contributor to its lipid-lowering effect [12]. Apigenin downregulates the expression of FGF21 (fibroblast growth factor 21, metabolic hormone; lipid lowering) while simultaneously upregulating CYP7A1 expression, thus exerting a lipid-lowering effect in a hyperlipidemic mouse model [51].

### 4.2. Secretion and Excretion

Flavonoids in Chrysanthemi Flos and Chrysanthemi Indici Flos may promote the secretion and excretion of bile acids through multiple pathways, including direct biliary excretion, regulation of efflux transporters, and nuclear receptor signaling pathways. Luteolin is mainly cleared via the biliary excretion pathway, with its 7-O-glucuronide metabolite being the predominant form in biliary excretion [52]. Studies have shown that after oral administration of luteolin, the cumulative biliary excretion in rats is about 2.05% of the administered dose within 36 h, and biliary excretion dominates the elimination of conjugated luteolin [53]. Apigenin is also cleared via the biliary excretion pathway. Following oral administration of Chrysanthemi Flos extract to rats, the cumulative biliary excretion of apigenin is about 6.34% of the administered dose within 36 h [54]. Secretion of kaempferol into bile is an important route of its excretion. Kaempferol is excreted via bile, and its enterohepatic triple circulation is mediated by the high biliary secretion rate in the enterohepatic axis; MRP2 is the major efflux transporter regulating the enterohepatic triple circulation of kaempferol [54]. In Bcrp^−^/^−^Mrp2^−^/^−^ double-knockout mice, the enterohepatic triple circulation of kaempferol is blocked, and its preventive and therapeutic effects on acute colitis are inhibited [55]. Kaempferol also exerts a prebiotic-like effect on bile acid metabolism by changing the gut microbiota: it upregulates tauro-conjugated bile acids by inhibiting BSH (bile salt hydrolase, bacterial deconjugation of bile acids) activity, which is associated with alterations in gut microbiota composition [56]. After absorption in the small intestine, quercetin undergoes phase II metabolism and is finally excreted by the liver into bile or by the kidneys into urine [57]. The intestinal excretion of quercetin varies depending on its chemical form; in perfusion experiments, about 52% of quercetin is secreted into the intestinal lumen in conjugated forms, suggesting that it also plays an important role in biliary efflux [54]. Hesperidin may regulate the expression of Mrp2 in the liver. Compared with the control group, Mrp2 expression in the liver of hesperidin-treated rats was significantly reduced to 37% of the control level, thus decreasing the biliary excretion of substrate drugs [58]. The oral bioavailability of hesperidin is lower than that of its aglycone, and the interplay between phase II enzymes and efflux transporters in the intestine reduces its relative oral bioavailability [59]. Chrysoeriol is rapidly metabolized in the isolated perfused rat liver and is partially excreted into bile in the form of glucuronide and sulfate conjugates [60]. Hyperoside upregulates the expression of nuclear receptors (Fxr and Shp) and the BSEP in mice with pyrrolizidine alkaloid (PA)-induced liver injury, thus regulating bile acid homeostasis and ameliorating PA-induced acute liver injury [61]. Luteolin and kaempferol were identified as potent OATP1B1 (organic anion transporting polypeptide 1B1, hepatic uptake transporter; inhibited by flavonoids) inhibitors (>50% inhibition) in OATP1B1-HEK293 cells, reducing OATP1B1-mediated methotrexate uptake and cytotoxicity and indicating a potential for flavonoid–drug interactions [62].

### 4.3. Enterohepatic Circulation

**Figure 3:**
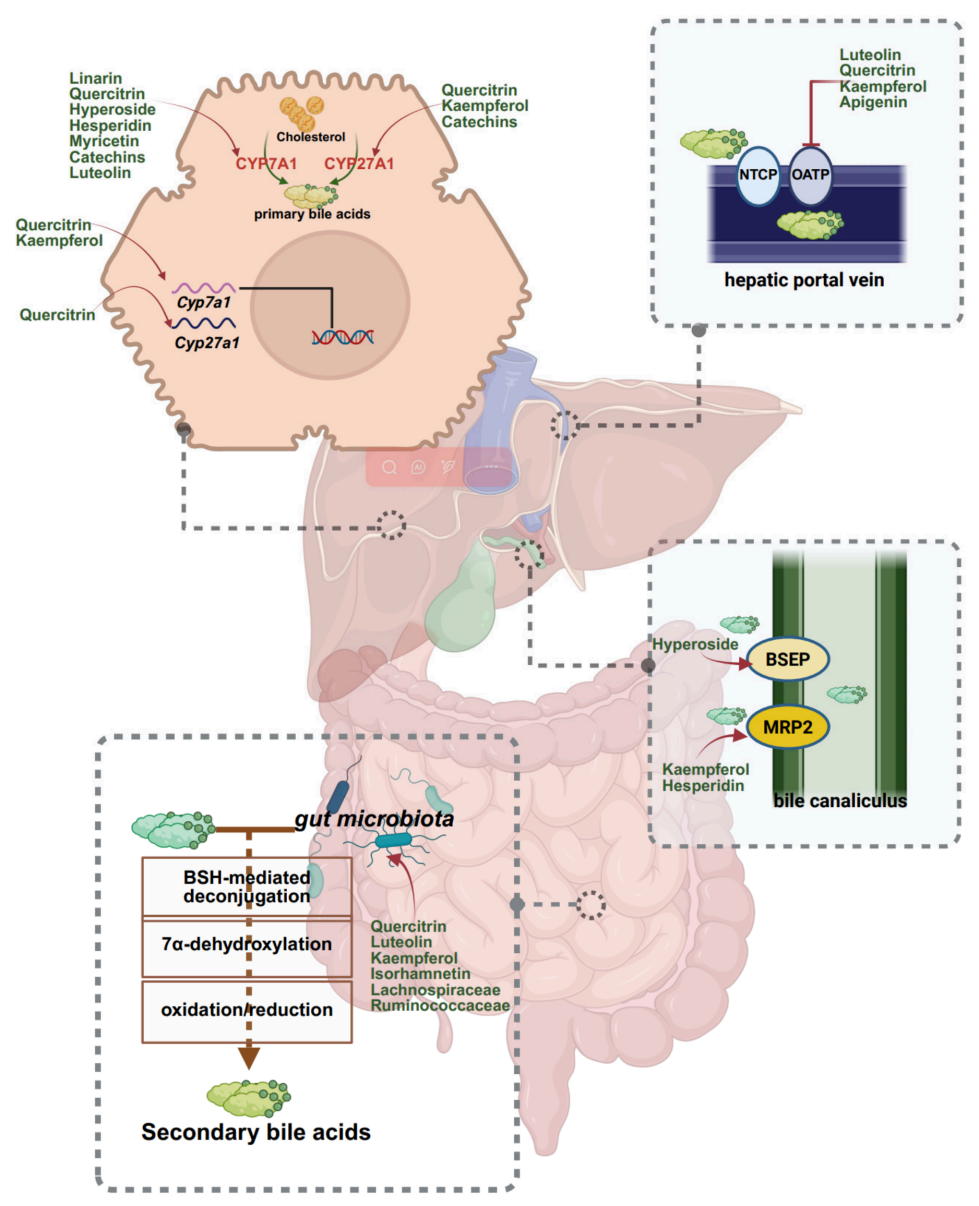
The mechanism of flavonoids in Chrysanthemi Flos and Chrysanthemi Indici Flos regulating bile acid metabolism. Created in BioRender. Jingchen, X. (2026) https://BioRender.com/3b5tf0t. This figure illustrates the multi-target regulatory network of Chrysanthemi flavonoids in bile acid synthesis, secretion, and enterohepatic circulation, emphasizing their synergistic effects on key enzymes, transporters, and gut microbiota.

Flavonoids in Chrysanthemi Flos and Chrysanthemi Indici Flos not only participate in the secretion and excretion of bile acids but also maintain bile acid homeostasis through multiple stages of the enterohepatic circulation, including intestinal reabsorption, hepatic reuptake, and microbial metabolism. The specific roles of each component are described below. Following oral administration of Chrysanthemi Flos extract, both luteolin and apigenin display a double-peak pattern in their plasma concentration–time curves, and biliary excretion confirmed the enterohepatic circulation of luteolin [53]. Also, luteolin-β-CD-MOF (a metal–organic framework complex synthesized by modifying luteolin with β-cyclodextrin to improve its bioavailability) exerts hepatoprotective effects by changing the gut microbiota and bile acid metabolism. This treatment significantly alters the gut microbiota composition and fecal bile acid profile, inhibits pathogenic bacteria, and reverses acetaminophen-induced liver injury [63]. In a chronic stress-induced rat model, quercetin activated the intestinal FXR/TGR5 signaling pathway, modified bile acid composition, and enhanced GLP-1 secretion, collectively contributing to improved glycemic and lipid homeostasis [64]. After oral administration of Chrysanthemi Flos extract to rats, apigenin similarly exhibited a double-peak pattern in the plasma concentration–time curve, suggesting its involvement in enterohepatic circulation [53]. Kaempferol is highly dependent on BCRP (breast cancer resistance protein, ATP-dependent efflux transporter; mediates flavonoid enterohepatic circulation)- and MRP2-mediated enterohepatic triple circulation. In Bcrp/Mrp2 double-knockout mice, the enterohepatic triple circulation of kaempferol was blocked. Deficiencies in BCRP and MRP2 led to impaired excretion of kaempferol and its phase II metabolites, resulting in elevated plasma levels [55]. Concurrently, kaempferol regulates gut microbiota composition and improves bile acid metabolism, thus alleviating steatosis and fibrosis in mouse models [65]. Also, as a prebiotic, kaempferol may have nutritional and therapeutic effects by regulating the interaction between bile acids and the gut microbiota, thus changing the enterohepatic circulation [56]. Isorhamnetin alleviates metabolic disorders-associated steatotic liver disease (MASLD) by reshaping the gut microbiota, increasing the relative abundance of Lachnospiraceae and Ruminococcaceae, promoting the levels of primary and conjugated bile acids, activating the liver-ileal FXR signaling axis, accelerating the enterohepatic bile acid cycle, and reducing dietary fat absorption [66]. Naringin and naringenin improve metabolic disorders-associated fatty liver disease by regulating the enterohepatic bile acid cycle [67]. Quercetin is transported across biological barriers in the enterohepatic–renal cycle by various transporters (e.g., SGLT1 (sodium–glucose linked transporter 1, Na^+^-dependent glucose transporter; may transport quercetin), OATPs (organic anion transporting polypeptides), and MRPs) and is finally secreted into bile via the hepatobiliary pathway or excreted by the kidneys [57]. Long-term intake of hesperetin-7-O-glucoside (the major metabolite of hesperidin) may regulate the gut microbiota and bile acid metabolism in mice [59]. Overall, flavonoids such as luteolin, apigenin, kaempferol, isorhamnetin, quercetin, hesperidin, and naringin found in Chrysanthemi Flos and Chrysanthemi Indici Flos regulate the enterohepatic circulation of bile acids through multiple mechanisms, including participation in bile excretion and reabsorption, changes in the gut microbiota and the FXR signaling axis, and transporter-mediated enterohepatic circulation (e.g., via BCRP and MRP2). These actions help maintain metabolic balance and provide evidence relevant to ameliorating cholestasis, liver injury, and metabolism-related diseases (Figure 3).

### 4.4. Bile Acid Receptors

**Figure 4:**
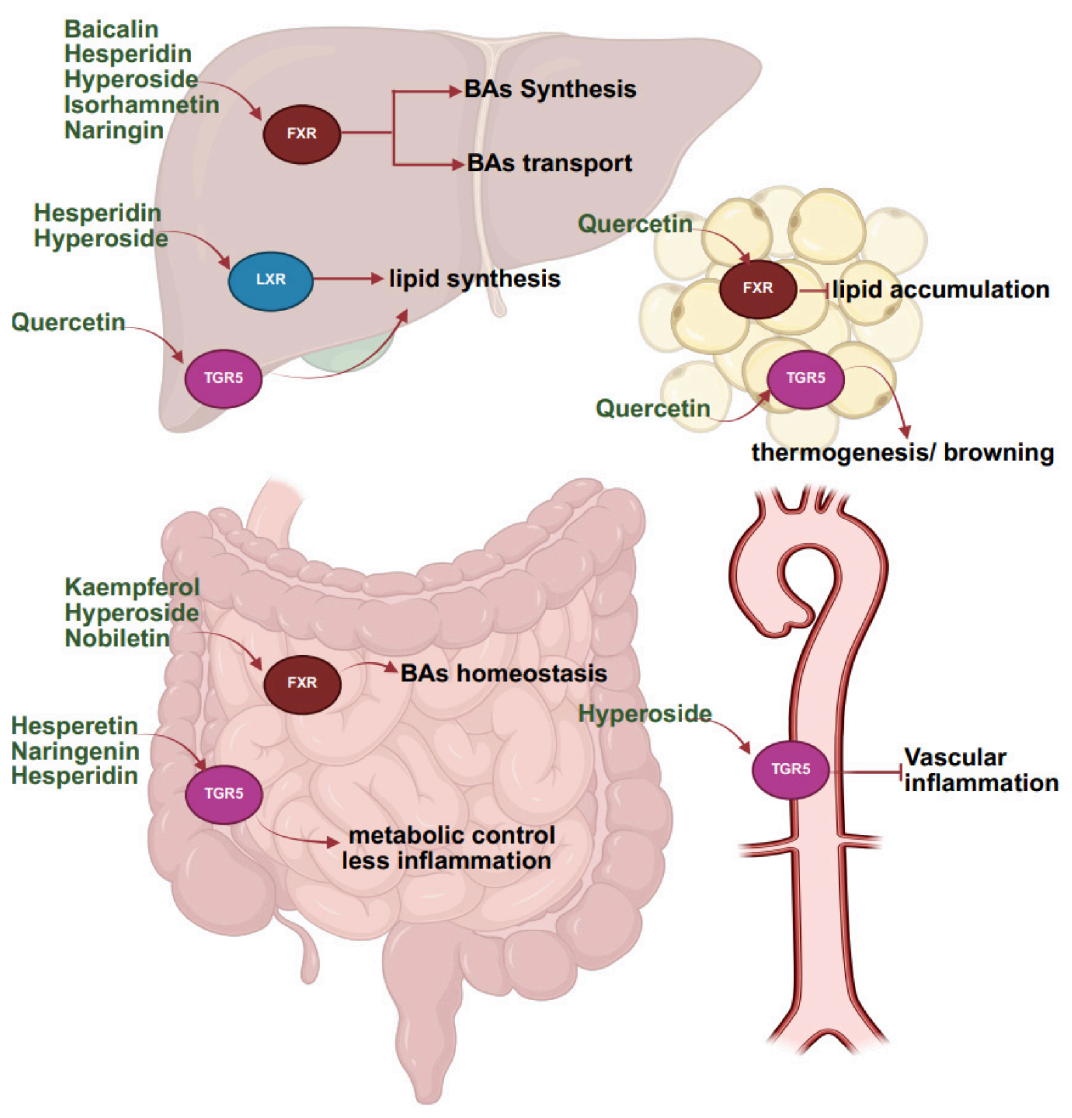
The mechanism of flavonoids in Chrysanthemi Flos and Chrysanthemi Indici Flos regulating bile acid receptors. Created in BioRender. Jingchen, X. (2026) https://BioRender.com/7ys6pwj. This figure depicts the receptor-level mechanisms (FXR, LXRα, TGR5) by which Chrysanthemi flavonoids modulate bile acid homeostasis across the liver, intestine, and adipose tissues, highlighting their potential in ameliorating metabolic and inflammatory diseases.

The flavonoids in Chrysanthemi Flos and Chrysanthemi Indici Flos (such as luteolin, apigenin, and linarin) mainly regulate bile acid metabolism and ameliorate cholestasis and lipid metabolism disorders in preclinical models by changing various nuclear bile acid receptors (particularly FXR) and related signaling pathways. The reported mechanisms are described below (Figure 4).

#### 4.4.1. Main Receptors and Pathways

##### Farnesoid X Receptor (FXR) Pathway

In a chronic-plus-binge alcohol-exposure mouse model, kaempferol selectively activates intestinal FXR, upregulates FGF15/19 (intestinal FXR-induced hormone; inhibits CYP7A1) expression, and suppresses hepatic bile acid synthesis, alleviating alcohol-induced liver injury [68]. This intestinal feedback mechanism differs from the hepatic CYP27A1 and NTCP expression changes reported in the MASH model [43]. Quercetin may regulate the gut microbiota species Akkermansia muciniphila and its metabolite ILA (indole-3-lactic acid), thus upregulating CYP8B1 expression through the FTO/m6A/YTHDF2 (YTH N6-methyladenosine RNA binding protein 2) signaling pathway, promoting CA synthesis, and finally activating FXR in adipose tissue to suppress lipid accumulation [40]. Baicalin upregulates FXR expression by activating the Sirt1/HNF-1α (Sirtuin 1/hepatocyte nuclear factor-1α, upregulates FXR) signaling pathway, thus changing bile acid synthesis, metabolism, and transport, restoring bile acid homeostasis, and inhibiting inflammatory responses, which may be relevant to estrogen-induced cholestasis (EIC) and related liver injury [4]. Dihydromyricetin (DHM) may reduce the production of CDCA by changing the gut microbiota, thus inhibiting FXR expression in intestinal L cells, promoting GLP-1 secretion, and finally improving insulin resistance and associated metabolic abnormalities [69]. In high-fat-diet-fed rats with MAFLD (metabolic dysfunction-associated fatty liver disease), hyperoside increased hepatic CYP7A1 and CYP27A1 protein levels together with FXR and LXRα expression [70]. It may also directly activate the intestinal FXR-FGF15 signaling pathway to suppress hepatic bile acid synthesis, maintain bile acid homeostasis in ALD (alcoholic liver disease) mice, and effectively alleviate chronic alcohol-induced liver injury [71]. Concurrently, by activating the hepatic FXR nuclear receptor, hyperoside inhibits CYP7A1 protein and gene expression while upregulating CYP27A1 expression, thus regulating bile acid metabolism [56]. Isorhamnetin may activate FXR and improve bile acid metabolism, reducing intestinal lipid absorption and inhibiting hepatic lipid synthesis [66]. Also, isorhamnetin regulates bile acid metabolism by upregulating FXR and BSEP while downregulating SLCO1B3 (solute carrier organic anion transporter family member 1B3, hepatic OATP1B3; mediates bile acid/drug uptake), thus alleviating hepatic bile acid accumulation, lowering serum and hepatic lipid levels, and attenuating liver injury, finally exerting protective effects against metabolic disorders-associated steatotic liver disease (MASLD) in mice [72]. Also, isorhamnetin may enhance FXR signaling through direct interaction with FXR, accelerating the enterohepatic bile acid cycle and reducing dietary fat absorption [66]. Naringin increases FXR expression in APAP-induced hepatotoxicity, suggesting possible relevance to counteracting APAP-induced liver and kidney toxicity [73].

##### Liver X Receptor α (LXRα) Pathway

Compounds such as naringin, poncirin, and neohesperidin regulate lipid and glucose metabolism by inhibiting the expression of liver X receptors (LXRα and LXRβ), thus ameliorating high-fat diet-induced obesity and related metabolic disorders [74]. Myricetin activates the LXR/RXR (retinoid X receptor) signaling pathway, upregulating the expression of cholesterol transport-related genes (such as CD36 (cluster of differentiation 36, fatty acid/oxLDL uptake), MSR1 (macrophage scavenger receptor 1, macrophage scavenger receptor; oxLDL uptake), and NR1H3 (nuclear receptor sub-family 1 group H member 3, LXRα gene; nuclear receptor)) and promoting cholesterol efflux from macrophages, thus regulating lipid metabolism and suggesting possible relevance to atherosclerosis [75]. Quercitrin and afzelin bind to LXRα and LXRβ (key hydrogen-bonding sites Thr302/Thr316) to activate the LXR signaling pathway, upregulating the expression of downstream target genes APOE (apolipoprotein E, lipoprotein; cholesterol transport/clearance) and ABCA1, thus regulating lipid metabolism (including cholesterol efflux and bile acid metabolism) and indicating their potential for further investigation in Alzheimer’s disease (AD) and inflammation-related diseases in preclinical models [76].

##### G Protein-Coupled Bile Acid Receptor 1 (TGR5)

In a chronic unpredictable mild stress (CUMS) rat model, pure quercetin significantly alleviated depressive-like behaviors and ameliorated glucose metabolic dysregulation by modifying colonic bile acid composition, activating the FXR/TGR5 pathway, and enhancing GLP-1 (glucagon-like peptide-1) secretion [64]. Quercetin promotes the production of non-12-hydroxy bile acids, particularly UDCA and LCA, by reshaping the gut microbiota (enriching *Lactobacillus* spp.). These bile acids act as TGR5 agonists, activating thermogenesis and browning in adipose tissue, thus increasing energy expenditure, and have been investigated in models of metabolic syndrome [77]. By modulating the gut bile acid profile, activating TGR5 and FXR signaling pathways, and promoting GLP-1 secretion, quercetin improves glucose and lipid metabolism disorders and has been investigated in models of chronic unpredictable mild stress (CUMS) [64]. By activating TGR5 and FXR signaling pathways, quercetin improves bile acid homeostasis, regulates lipid metabolism, and reduces hepatic fat accumulation, suggesting possible relevance to type 2 diabetes mellitus-induced metabolic disorders-associated steatotic liver disease (T2DM-induced MASLD) [78]. In a study using spontaneously hypertensive rats, hyperoside, a prominent bioactive within hawthorn, independently reduced blood pressure by regulating primary bile acid biosynthesis and inflammation-related targets, including FXR, TGR5, and RAS (renin-angiotensin system, blood pressure/fluid balance; vascular inflammation) components [79]. Hesperetin and naringenin could directly activate TGR5, thereby increasing cAMP/Ca^2+^ signaling and GLP-1 secretion and improving glucose tolerance, obesity and hyperglycemia in diet-induced diabetic mice [80]. In diabetic mice, hesperidin increased intestinal TGR5/cAMP/CREB (cAMP response element-binding protein)/GLP-1 signaling, concomitant with improved glycemia, enhanced insulin and C-peptide levels, reduced β-cell apoptosis and promotion of α-to-β cell transdifferentiation [81].

##### Other Nuclear Receptors

Hesperidin inhibits hepatic ACCα (acetyl-CoA carboxylase α, lipogenesis; acetyl-CoA → malonyl-CoA) and FAS (fatty acid synthase) and enhances antioxidant and anti-inflammatory effects [34]; hyperoside upregulates UDP-glucuronosyltransferase (glucuronidates bile acids; increases hydrophilicity), thus increasing hydrophilic conjugated bile acids [68].

Overall, the flavonoids in Chrysanthemi Flos and Chrysanthemi Indici Flos synergistically regulate bile acid metabolism through multiple receptors and pathways, with FXR as the central target while also engaging receptors such as LXRα and TGR5. These actions collectively achieve choleretic and detoxification effects, promoting bile acid excretion and inhibiting toxic accumulation; lipid-lowering and metabolic regulation, facilitating cholesterol conversion and excretion; and maintenance of gut–liver axis homeostasis, suppressing excessive bile acid synthesis through feedback regulation via intestinal receptors and the gut microbiota. These mechanisms provide important receptor-level evidence for the application of Chrysanthemi Flos and Chrysanthemi Indici Flos in the prevention and treatment of cholestatic liver disease, hypercholesterolemia, and metabolism-related liver injury.

## 5. Discussion

Chrysanthemi Flos and Chrysanthemi Indici Flos, as traditional Chinese medicinal plants used for both medicinal and dietary purposes, have attracted significant attention for their pharmacological activity. Modern research indicates that the flavonoid compounds they contain are important contributors to their multiple health benefits, including antibacterial, anti-inflammatory, antioxidant, lipid-lowering, blood pressure-lowering, and antitumor effects [1,7,8,9,10]. To date, at least 52 flavonoid compounds have been identified in Chrysanthemi Flos and Chrysanthemi Indici Flos, encompassing various structural types such as flavones, flavanols, dihydroflavones, isoflavones, catechins, and their glycosides. Of these, 38 are derived from Chrysanthemi Flos and 16 from Chrysanthemi Indici Flos (Table 1, Figure 1). In recent years, growing evidence indicates that bile acid metabolism is one of the main mechanisms through which flavonoids exert systemic metabolic regulatory effects. For example, total flavonoids from Chrysanthemi Flos significantly improve lipid levels in mice with hypercholesterolemia [82], while linarin from Chrysanthemi Indici Flos alleviates liver damage by regulating cholesterol metabolism. However, previous reviews have mainly focused on the direct anti-inflammatory and antioxidant effects of flavonoids, whereas a systematic summary of their indirect regulation of metabolic and immune balance via the bile acid pathway remains lacking. Therefore, this article systematically reviews the molecular mechanisms by which flavonoid compounds in Chrysanthemi Flos and Chrysanthemi Indici Flos regulate bile acid synthesis, binding, transport, excretion, and the enterohepatic circulation, to provide a theoretical basis for the in-depth development and clinical translation of these compounds.

Bile acid metabolism has a broad impact on the onset and progression of various diseases. In metabolic diseases, bile acids are intricately linked with obesity [40], type 2 diabetes [64], and nonalcoholic fatty liver disease [83]. In liver diseases, dysregulation of bile acid metabolism may lead to cholestatic liver injury [4], alcoholic liver disease [71], drug-induced liver injury, and liver fibrosis. In the cardiovascular field, bile acid metabolism regulates the conversion of cholesterol to bile acids, thus influencing atherosclerosis [75] and hyperlipidemia [83]. Also, bile acid signaling is involved in colorectal cancer, ulcerative colitis [84], and other intestinal disorders such as constipation [85], as well as renal pathologies including diabetic nephropathy and drug-induced kidney injury [72], while also exerting anti-inflammatory and immunomodulatory effects via the TGR5 receptor. Through main signaling molecules such as the FXR, the G protein-coupled receptor TGR5, and the LXRα, bile acids regulate glucose and lipid metabolism, energy balance, and bidirectional communication between the gut microbiota and the host, influencing inflammatory responses, intestinal barrier function, and immune cell activation. As a result, they play a central regulatory role in pathological processes such as nonalcoholic steatohepatitis [82], cholestatic cirrhosis [4], inflammatory bowel disease [86], and atherosclerotic cardiovascular disease, supporting the view that bile acid metabolism is a common link among diseases in several organ systems.

The available preclinical studies suggest that the flavonoids found in Chrysanthemi Flos and Chrysanthemi Indici Flos may protect against metabolic diseases, liver damage, and inflammation-related conditions in animal and cell models by intervening in multiple stages of bile acid metabolism, including synthesis, conjugation, transport, excretion, and the enterohepatic circulation. Their regulatory mechanisms are characterized by multi-target interactions, interaction with the gut–liver axis, and integration of receptor networks. Hyperoside may also activate LXRα to enhance CYP7A1 transcription, forming a feedback regulatory network [70]. Regarding the gut–liver axis and gut microbiota, isorhamnetin increases the abundance of beneficial bacteria and promotes the accumulation of conjugated bile acids to accelerate the enterohepatic circulation [66], while kaempferol exerts a prebiotic-like effect by inhibiting the activity of bile salt hydrolases and upregulating taurine-conjugated bile acids [56], thus achieving systemic metabolic regulation through “gut–liver communication”. Bile acid receptors such as TGR5 are also involved in this process. Secondary bile acids activate TGR5 to promote GLP-1 secretion and inhibit the NF-κB (nuclear factor kappa-B) pathway, thus exerting anti-inflammatory effects [87]. Quercetin regulates hepatic bile acid metabolism by modulating the expression of FXR and BSEP [88]. Overall, the flavonoids in Chrysanthemi Flos and Chrysanthemi Indici Flos form a molecular network that regulates bile acid metabolism by integrating multiple processes, including bile acid synthesis, transport, excretion, and the enterohepatic circulation. This may help explain their traditional medicinal effects, such as “cleansing the liver and promoting bile secretion” and “lowering lipids and improving vision,” and may guide the development of natural medicines targeting the bile acid pathway.

Disease context provides a framework for interpreting these apparently opposing effects, but should not be used to explain away incorrect attribution or unsupported causal claims. Where cholesterol disposal is the principal metabolic concern, greater conversion to bile acids may contribute to lipid lowering; where bile acid handling is disrupted during liver injury, restraining synthesis may help reduce the burden on hepatocytes. The contrasting findings for hyperoside in NAFLD rats and emodin-injured cells, and for kaempferol in NASH (nonalcoholic steatohepatitis) and alcoholic liver disease, are consistent with this context-dependent interpretation [42,44,68,70]. Tissue specificity also matters: intestinal FXR-FGF15 feedback and hepatic receptor expression are different readouts, and increased hepatic FXR abundance alone does not determine the net direction of CYP7A1 regulation [68,70]. Moreover, CYP27A1-associated pathway changes can coexist with reduced CYP7A1 expression [56]; they should not be described simply as simultaneous stimulation and inhibition of total synthesis. Bile acid pool size and composition also reflect uptake, export, intestinal reabsorption, and microbial transformation, rather than synthesis alone. Disease state is therefore one plausible determinant alongside compound identity, dose, exposure duration, species, and baseline bile acid composition. Because these studies were not matched comparisons of the same intervention across diseases, they do not prove that a flavonoid automatically switches direction to normalize bile acid metabolism. Future studies should combine synthesis-flux measurements with enzyme activity, tissue-specific receptor perturbation, and bile acid profiling to distinguish these possibilities.

Overall, the flavonoid components in Chrysanthemi Flos and Chrysanthemi Indici Flos may regulate bile acid metabolism through several targets and may help prevent or treat metabolic diseases and liver injury. However, existing research still has certain limitations. Several areas require further investigation. First, the regulatory roles of most individual compounds (such as acacetin, eriodictyol, and astilbin) on bile acid receptors (FXR, TGR5, LXRα, etc.) and metabolic enzymes (CYP7A1, CYP27A1, BSEP, etc.) remain unclear. Future studies should examine their mechanisms involving several targets and structure–activity relationships through high-throughput screening techniques such as molecular docking, surface plasmon resonance, and reporter gene assays. Second, the in vivo concentrations of these flavonoids in specific target tissues (such as hepatocytes and enterocytes) and their comparison with effective concentrations in vitro remain largely unexplored. Future studies should focus on determining the in vivo distribution and bioavailability of these compounds to bridge this gap between preclinical findings and clinical application. Furthermore, in addition to focusing on individual flavonoid monomers such as quercetin and hyperoside, future research should further integrate multiomics methods, including network pharmacology and metabolomics, to clarify the molecular basis and synergistic interactions of interventions that combine several components on bile acid metabolism within the complex extract systems of Chrysanthemi Flos and Chrysanthemi Indici Flos. Also, existing evidence is derived mainly from cell and animal studies, and there is a lack of high-quality randomized controlled clinical trials. Future work should include integrated pharmacokinetic–pharmacodynamic studies targeting patients with cholestatic liver disease, nonalcoholic fatty liver disease, and hyperlipidemia, using Chrysanthemi Flos, total flavonoids from Chrysanthemi Flos and Chrysanthemi Indici Flos, or representative monomers (such as luteolin, quercetin, and linarin) as interventions, to clarify their effects on bile acid metabolism in humans and their safety. Such studies could support the development of targeted natural medicines based on the bile acid pathway.

## 6. Conclusions

Chrysanthemi Flos and Chrysanthemi Indici Flos contain structurally diverse flavonoids that may regulate bile acid homeostasis through a coordinated, FXR-centered regulatory network. Chrysanthemi flavonoids regulate bile acid metabolism at multiple interconnected levels by modulating FXR-related signaling and key enzymes and transporters involved in bile acid synthesis and excretion. They also affect TGR5 and LXRα signaling, gut microbial biotransformation, and enterohepatic circulation. Through this integrated gut–liver regulatory network, these flavonoids may rebalance the bile acid pool and thereby contribute to the regulation of lipid and glucose metabolism, inflammatory responses, and liver injury. This mechanism provides a potential molecular basis linking the traditional use of Chrysanthemi Flos and Chrysanthemi Indici Flos with their reported hepatoprotective and metabolic effects. Nevertheless, most available evidence remains preclinical, and direct target-specific and clinical evidence is limited. Future studies integrating bile acid metabolomics, pharmacokinetics, gut microbiome analysis, target-specific validation, and clinical investigations are required to establish causal relationships and determine the translational relevance of this proposed regulatory network.

## Figures and Tables

**Table 1:** Flavonoids in Chrysanthemi Flos and Chrysanthemi Indici Flos.

NO	Classification	Compound	Molecular Formula	Distribution	References
1	Flavone	Chrysoeriol	C_16_H_12_O_6_	a&b	[16,17]
2	Flavone	Luteolin	C_15_H_10_O_6_	a&b	[16]
3	Flavone	Apigenin	C_15_H_10_O_5_	a&b	[16,17]
4	Flavone	Acacetin	C_16_H_12_O_5_	a&b	[16,17]
5	Flavone	Eupatilin	C_18_H_16_O_7_	a&b	[16,18]
6	Flavone	5,7,3′,4′-tetrahydroxy-6,5′-dimethexy-flavone	C_17_H_14_O_8_	b	[19]
7	Flavone	Cirsimaritin	C_17_H_14_O_7_	b	[20]
8	Flavone	Chrysin	C_15_H_10_O_4_	b	[20]
9	Flavone	5,7,4′-Trihydroxy-3′,5′-dimethoxyflavone (Tricin)	C_17_H_14_O_7_	b	[21]
10	Flavone	5,3′,4′-Trihydroxy-6,7-dimethoxyflavone	C_17_H_14_O_7_	b	[21]
11	Isoflavone	Prunetin	C_16_H_12_O_5_	b	[22]
12	Flavanone	Hesperetin	C_16_H_14_O_6_	a	[22]
13	Flavanone	Eriodictyol	C_15_H_12_O_6_	a&b	[22,23]
14	Flavanone	Naringenin	C_15_H_12_O_5_	b	[20]
15	Flavonol	Kaempferol	C_15_H_10_O_6_	a&b	[16,17]
16	Flavonol	Quercetin	C_15_H_10_O_7_	a&b	[24]
17	Flavonol	Myricetin	C_15_H_10_O_8_	b	[22]
18	Flavonol	Quercetin-3-O-rutinoside	C_2__7_H_30_O_16_	b	[22]
19	Flavonol	Isorhamnetin	C_16_H_12_O_7_	b	[22]
20	Flavonol	Myricitrin	C_21_H_20_O_12_	b	[20]
21	Flavonol	Quercitrin	C_21_H_20_O_11_	b	[20]
22	Flavonol	Hyperoside	C_21_H_20_O_12_	b	[20]
23	Flavonol	Astragalin	C_21_H_20_O_11_	b	[20]
24	Catechin (Flavan-3-ol)	Catechin	C_15_H_14_O_6_	a&b	[17,20]
25	Dihydroflavonol	Astilbin	C_21_H_22_O_11_	b	[20]
26	Dihydroflavonol	Taxifolin	C_15_H_12_O_7_	b	[20]
27	Dihydroflavonol	Aromadendrin	C_15_H_12_O_6_	b	[20]
28	Flavone glycoside	Diosmetin-7-O-beta-D-glucopyranoside	C_22_H_22_O_11_	a&b	[16,25]
29	Flavone glycoside	Luteolin-7-O-β-D-glucopyranoside	C_21_H_20_O_11_	a&b	[16,25]
30	Flavone glycoside	Acacetin-7-O-β-D-glucopyranoside	C_22_H_22_O_10_	a&b	[16,26]
31	Flavone glycoside	Acacetin-7-O-β-D-glucopyranosyl(6 → 1)-α-L-rhamnopyranoside	C_33_H_40_O_19_	a	[16]
32	Flavone glycoside	Acacetin-7-O-(6″-O-acetyl)-β-D-glucopyranoside	C_24_H_24_O_11_	a&b	[16,26]
33	Flavone glycoside	Luteolin-7-O-rutinoside	C_27_H_30_O_15_	a&b	[24,25]
34	Flavone glycoside	Luteoloside (Luteolin-7-O-glucoside)	C_21_H_20_O_11_	a	[24]
35	Flavone glycoside	Apigenin-7-O-rutinoside	C_27_H_30_O_14_	a	[24]
36	Flavone glycoside	Apigenin-7-O-glucoside	C_21_H_20_O_10_	a&b	[24,25]
37	Flavone glycoside	Apigenin-7-O-glucuronide	C_21_H_18_O_11_	a	[24]
38	Flavone glycoside	Luteolin-7-O-(6″-malonyl)-glucopyranoside	C_24_H_22_O_14_	a	[24]
39	Flavone glycoside	Apigenin-7-O-(6″-malonyl)-glucopyranoside	C_24_H_22_O_13_	a	[24]
40	Flavone glycoside	Acacetin-7-O-rutinoside	C_28_H_32_O_14_	a&b	[17,24]
41	Flavone glycoside	Acacetin-7-O-glucoside	C_22_H_22_O_10_	a	[24]
42	Flavone glycoside	Acacetin-7-O-β-D-(3″-acetyl)-glucopyranoside	C_24_H_24_O_11_	a	[27]
43	Flavone glycoside	Luteolin-7-O-β-D-(6″-acetyl)-glucopyranoside	C_23_H_22_O_12_	a	[27]
44	Flavone glycoside	Hesperetin-7-O-β-D-glucopyranoside	C_22_H_24_O_11_	a	[27]
45	Flavone glycoside	Acacetin-7-O-β-D-glucopyranoside	C_22_H_24_O_10_	a	[27]
46	Flavone glycoside	Apigenin-7-O-β-D-glucopyranoside	C_21_H_20_O_10_	a	[27]
47	Flavone glycoside	Luteolin-7-O-β-D-glucopyranoside	C_21_H_20_O_11_	a	[27]
48	Flavone glycoside	Luteolin-7-O-glucuronide	C_21_H_18_O_12_	a	[22]
49	Flavone glycoside	Qucertin-7-O-β-D-glucopyranoside	C_21_H_20_O_12_	b	[19]
50	Isoflavone glycoside	Acacetin-7-O-(6″-malonyl)-glucopyranoside	C_25_H_24_O_13_	a	[24]
51	Flavanone glycoside	Hesperidin	C_28_H_34_O_15_	a	[27]
52	Flavanone glycoside	Naringin	C_27_H_32_O_14_	b	[20]

Note: a = Chrysanthemi Flos; b = Chrysanthemi Indici Flos; a&b = detected in both plant.

## Data Availability

No new data were created or analyzed in this study. Data sharing is not applicable to this article.
